# Mandarin biochar-CO-TETA was utilized for Acid Red 73 dye adsorption from water, and its isotherm and kinetic studies were investigated

**DOI:** 10.1038/s41598-024-62870-x

**Published:** 2024-06-06

**Authors:** Ahmed Eleryan, Eda Keleş Güner, Mohamed Hassaan, Mohamed A. El-Nemr, Safaa Ragab, Ahmed El Nemr

**Affiliations:** 1 Environment Division, National Institute of Oceanography and Fisheries (NIOF), Kayet Bey, Elanfoushy, Alexandria, Egypt; 2 Uzumlu Vocational School, Department of Property and Security, Erzincan Binali Yıldırım University, Erzincan, Turkey; 3 Department of Chemical Engineering, Faculty of Engineering, Minia University, Minia, 61519 Egypt

**Keywords:** Biochar, Mandarin peels, Acid Red 73 dye, Adsorption isotherms, Kinetic parameters, Pollution remediation, Chemical engineering

## Abstract

Environmental pollution is a major issue today due to the release of dyestuff waste into the environment through industrial wastewater. There is a need for affordable and effective adsorbents to remove harmful dyes from industrial waste. In this study, Mandarin biochar-CO-TETA (MBCOT) adsorbent was prepared and used to remove Acid Red 73 (AR73) dye from aqueous solutions. The efficiency of dye removal was influenced by various factors such as solution pH, contact time, initial AR73 dye concentration, and MBCOT dosage. All experiments were conducted at 25 ± 2 °C, and the optimal pH was determined to be 1.5. The optimal conditions for dye removal were found to be an AR73 dye concentration of 100 mg/L, an MBCOT dosage of 1.5 g/L, and a contact time of 150 min, resulting in a 98.08% removal rate. Various models such as pseudo-first-order (PFO), pseudo-second-order (PSO), film diffusion (FD), and intraparticle diffusion (IPD) were used to determine the adsorption kinetics of AR73 dye onto MBCOT. The results showed that the PSO model best explains the AR73 dye adsorption. Furthermore, Langmuir and Freundlich's isotherm models were studied to explain the adsorption mechanism using experimental data. The adsorption capacities at equilibrium (qe) in eliminating AR73 dye varied from 92.05 to 32.15, 128.9 to 65.39, 129.25 to 91.69, 123.73 to 111.77, and 130.54 to 125.01 mg/g. The maximum adsorption capacity (*Q*_m_) was found to be 140.85 mg/g. In conclusion, this study demonstrates that biochar produced from mandarin peels has the potential to be an effective and promising adsorbent for removing AR73 dye from water.

## Introduction

The rapid depletion of water resources is a growing concern due to urbanization, population growth, industrial expansion, and unregulated resource usage. Many industries, including textile^1^, cosmetics^2^, plastics^3^, pharmaceuticals^4^, food processing^5^, and paint^6^, use dyes, heavy metals and other waste materials that pose a threat to the environment, particularly water resources^7^. This pollution can cause harm to people's eyes, skin, neurological, cardiovascular, and gastrointestinal systems, due to the presence of toxic compounds in water^8^.

Acid Red 73 (AR73) dye is a commonly used organic azo dye with a diazo and di-sulfonic structure. It is popular due to its affordability, good dyeing performance, and minimal fading. However, it is also a persistent organic pollutant that can cause harm to aquatic ecosystems and humans. It is known to be toxic, carcinogenic, and mutagenic. Due to these concerns, society is becoming increasingly worried about its use^9^. AR73 dye is bio-resistant because it has stable chromophore structures that contain –N=N– units, benzene rings, and naphthalene rings^10^.

The primary objective of wastewater treatment is to remove all pollutants to a satisfactory level by utilizing effective and cost-efficient techniques that provide recoverable water. The methods utilized to eliminate these pollutants from wastewater essentially comprise physical adsorption^11,12^, membrane filtration^13^, ion exchange^14^, settling ponds^15^, oxidation^16^, chemical coagulation-flocculation^17^, photocatalytic^18^, ozone and Fenton^19^, chemical oxidation processes^20^, electrochemical^21^, irradiation^22^, and biological enzyme-assisted^23^, bacteria-assisted^24^, fungal-assisted^25^ treatment methods.

Adsorption technology has gained significant attention among the existing purification techniques due to its simplicity, availability, and high efficiency^26–28^. However, the commonly used adsorbents have certain drawbacks, such as their high cost, poor adsorption, and sluggish response rates^29^. Adsorbents with a wide surface area and sufficient active sites often exhibit strong adsorption and a high ability to remove contaminants, as demonstrated by activated carbon^30^.

In general, activated carbon (AC) is preferable in adsorption procedures^31–33^. However, the cost of activated carbon is high, and there is a desire to obtain AC from cheaper sources. Therefore, using biomass-based adsorbents in the removal of dyestuffs from wastewater has become popular as it reduces some limitations such as operational efficiency, cost, energy, and harmful consequences^34^. For this reason, activated biomass-based treatments have recently gained popularity, demonstrating the potential to eliminate dyes and wastewater^35^.

The application of biochars in water purification for colorant removal can be improved by increasing the quantity and diversity of functional groups on their surfaces^36^. This can be achieved by modifying their surfaces chemically. There are several ways to improve the adsorption capacity of biochar, including metal impregnation, oxidation, carbon surface activation, and nanoscale formation^37^. The addition of amino groups to the surface of biochar can enhance its adsorption capacity. Applying different acids (HNO_3_, H_2_SO_4_, or H_3_PO_4_), bases (KOH, NaOH), or oxidizing reagents (O_3_, H_2_O_2_, NH_3_^.^H_2_O, KMnO_4_, or (NH_4_)_2_S_2_O_8_) to biochar can enhance the number of functional groups present in it^38–40^. Biochars that incorporate nanometals have been shown in several studies to have excellent thermal stability, enhanced oxidation resistance, increased surface area, and expanded adsorption sites. The most often used reducing agents are FeSO_4_, H_2_, NH_3_H2O, Na_2_SO_3_, and aniline^35,41^. Nowadays, active substances for adsorption studies are obtained from agricultural wastes. The reasons for obtaining carbon from agricultural waste are the high price of commercial activated carbons, their organic nature, easy availability, non-threatening nature to health, and cost-effectiveness. Since agricultural wastes used as activated carbon give excellent results in purifying water resources, these studies have gained significant momentum. These include the peels or seeds of many agricultural materials such as oranges^42,43^, mandarin^44,45^, lemons^46^, grapefruits^47^, pomegranates^48^, bananas^49^, apricots^50^, eggs^51^, walnuts^52^, peanuts^53^, hazelnuts^54^, rice^55^, olives^56^ and Palm shell^57^.

Mandarin is a popular citrus fruit that belongs to the *Citrus reticulata* species in the Rutaceae family. It grows in temperate climates and is known for its seedless structure, sweetness, and ease of hand peeling. Mandarin is a rich source of different chemical components such as antioxidants, dietary fibre, essential oils, flavonoids, carotenoids, and vitamin C. Due to these components, it has various medicinal properties such as reducing oxidative stress, promoting digestive health and immunity, lowering blood pressure, and even anti-cancer properties^58^.

The Food and Agriculture Organisation of the United Nations issued figures in 2021 that predicted the annual citrus output to be 80 million tonnes. China is the top producer, followed by Turkey in second place, Brazil in third place, Egypt, Japan, Spain, South Korea, and Italy^59^. After being utilized, peels from mandarin fruits are discarded into the environment, accounting for 8–14% of the fruit used to make fruit juice. The fertilizer, solid fuel, animal feed, and cosmetic sectors are the main companies that use them^60,61^. Due to mandarin being used so widely, a large volume of fruit peel ends up as biomass waste. Because mandarin peels include elements of organic carbon (hemicellulose, cellulose, lignin, and pectin) in their structure, they can be pyrolyzed to produce ecologically benign biochars^62,63^. High adsorption capacity materials may be produced using this method.

The study aimed to investigate the effectiveness of MBCOT produced from mandarin peel waste as an adsorbent for removing AR73 dye from water. The process involved dehydrating the mandarin peel waste with 85% H_2_SO_4_, oxidizing it with H_2_O_2_, and aminating it with triethylenetetramine. The resulting product, known as Mandarin biochar-CO-TETA (MBCOT), was tested under different conditions such as starting dye concentration, pH level, duration of interaction between MBCOT and AR73 dye, and MBCOT dose. To determine the maximum adsorption capacity (*Q*_m_) and the adsorption process for the AR73 dye adsorption by MBCOT, the kinetic and isotherm models were used. This study offers a promising solution for utilizing agricultural waste, such as mandarin peel, to produce an effective and inexpensive adsorbent for removing harmful dyes from water. To the authors' knowledge, this is the first time to use MBCOT for AR73 dye removal.

## Materials and methods

### Materials

**Figure 1:**
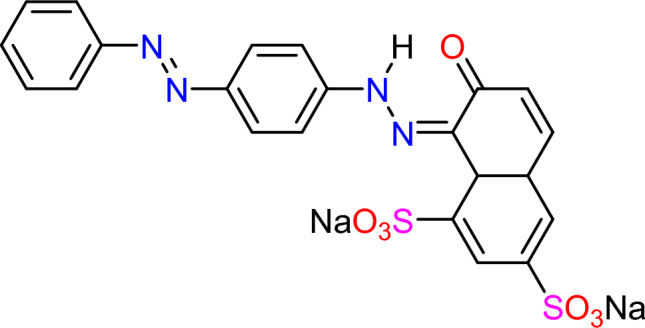
The AR73 dye chemical structure (MF: C_22_H_14_N_4_Na_2_O_7_S_2_) (MW: 556.48 g/mol) (C.I.27290) (CAS number: 5413–75-2).

For MBCOT production, Mandarin (*Citrus reticulata*) peels were obtained from a local market (Alexandria, Egypt). Sigma Aldrich provided the Sulfuric acid (H_2_SO_4_, M.W. = 98.07 g, 99%), Triethylenetetramine (TETA) and AR73 dye (Fig. 1). One liter of distilled water was used to dissolve one gram of dye to prepare the AR73 dye standard stock solution.

### Fabrication of MBCOT

Mandarin orange peels (*Citrus reticulata*) were thoroughly cleaned with water multiple times to remove any dust and then dried at 115 °C for 72 h. The dried peels were pulverized and crushed for this recipe. The experiment involved boiling 250 g of crushed mandarin peels in 2L of 85% H_2_SO_4_ for 5 h at ~ 240 °C in a reflux system. The peels were then diluted with deionized water, filtered, and repeatedly rinsed with water until the resulting filtrate reached a neutral consistency. After that, they were washed with ethanol, dried at 120 °C for the entire night, and weighed to produce 105 g of biomass-based biosorbent biochar made of mandarin. This biosorbent was transferred to 400 mL of 50% H_2_O_2_ and then heated at 70 °C for 60 min, producing 70 g of mandarin biochar-COH biosorbent after filtration, cleaning, and drying at 115 °C overnight. The mandarin biochar-COH biosorbent (50 g) was refluxed (280 °C) in 120 mL of TETA solution for two hours, resulting in 62 g of the product after filtration, and washing twice with ethanol and distilled water. The synthesis procedures of MBCOT are illustrated in Fig. S1. The product was labelled MBCOT and the characterization was found to be similart to that previously reported by us^61,64^, and all the characterizations data were reported in the supplementary data as Figs. S2–S15.

### Batch adsorption procedure

A stock solution of AR73 dye was created by dissolving 1.0 g of the dye in one litre of distilled water, resulting in a concentration of 1000 mg/L. The standard solution used for calibration and absorption experiments was produced by diluting the stock solution to the required concentrations. The absorption potential, isotherm and kinetic properties of MBCOT were determined through bulk adsorption studies. A series of 300 mL Erlenmeyer flasks were filled with 100 mL of AR73 dye solution at concentrations of 50, 100, 150, 200, and 250 mg/L, along with various doses of MBCOT at 0.50, 0.75, 1.0, 1.25, and 1.5 g/L at 25 °C. The flasks were shaken at 200 rpm for a predetermined amount of time, with the pH of the solution adjusted using 0.1 M HCl or 0.1 M NaOH, as required. Using spectrophotometry at *λ*_max_ = 518 nm, the concentration of the AR73 dye was determined after a 0.5 mL sample of the solution was taken out at different times until equilibrium was established. All experiments were repeated three times and the mean values were reported and the standard deviation was ≤ 2.5. The average data for kinetic and isotherm investigations was used to conduct the adsorption experiments three times. Using Eq. (1), the adsorption capacity at equilibrium (*q*_e_) was obtained.

1 $${q}_{t}=\frac{{C}_{o}-{C}_{t}}{W}\times V$$

where the adsorbent's ability to adsorb AR73 dye from a solution at a specific time is known as its capacity for adsorption (*q*_t_, mg/g). *C*_0_ (mg/L) is the initial AR73 dye concentration; *C*_t_ (mg/L) is the residual AR73 dye concentration after a certain period. Equation (2) may be used to calculate the percentage of AR73 dye removed from water.

2 $$Removal \left(\%\right)= \frac{{(C}_{o}-{C}_{t})}{{C}_{o}}\times 10$$

To assess how pH affects the adsorption of AR73 dye by MBCOT, 0.15 g of MBCOT was mixed with 100 mL of 100 mg/L initial AR73 dye concentration. Different initial pH values ranging from 1.5 to 12.2 were studied, and to modify the pH values, 0.1 M HCl or NaOH solutions were used. Before the mixture was analyzed for AR73 dye concentration, it was stirred at 200 rpm for 2.5 h at 25 °C. Isotherm studies were conducted by shaking 100 mL of solutions with varying starting concentrations of AR73 dye (50, 100, 150, 200, and 250 mg/L) at 200 rpm for three hours at 25 °C with different quantities of MBCOT (0.5 to 1.5 g/L). To examine the effects of MBCOT dose and contact duration on AR73 dye adsorption, 100 mL of AR73 dye was shaken with different doses of MBCOT (0.50, 0.75, 1.0, 1.25, and 1.5 g/L) at 25 °C.

*Author statement for the use of plants*. In this work, experimental research and field studies were conducted on the waste of mandarin orange peels (*Citrus reticulata*). The collection of plant peel waste was carried out in compliance with relevant institutional, national, and international guidelines and legislation.

## Results and discussion

### Adsorption of AR73 Dye on MBCOT

#### Effect of pH

**Figure 2:**
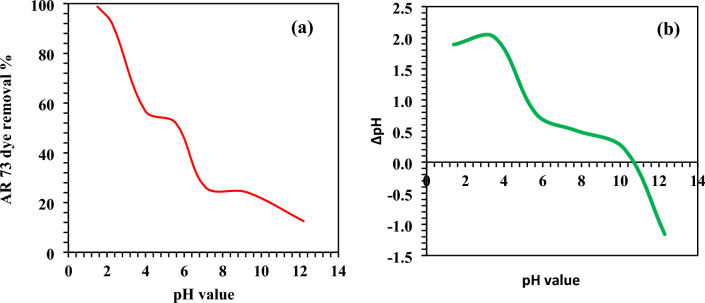
(**a**) Effect of pH on percentage removal of AR73 dye using MBCOT adsorbent; (**b**) *Δ*pH against pH plot (temperature = 25 ± 2 °C, starting AR73 dye concentration = 100 mg/L, MBCOT dosage = 1.5 g/L).

The adsorption of biochar is significantly influenced by the pH of a solution. This is because it has an impact on the surface's carboxyl, hydroxyl, and amino groups. Using 1.5 g/L MBCOT adsorbent and 100 mg/L starting dye concentration at room temperature, the adsorption of AR73 dye was tested at various pH values ranging from 1.5 to 12.2 for two hours to study this impact. According to the results, which are displayed in Fig. 2a, the highest removal of AR73 dye (98.8%) happened at pH 1.5 while employing MBCOT. The adsorption rate dropped from 98.8% to 12.5% when the pH value progressively rose from 1.5 to 12.2. Between pH 1.5 and pH 7.12, the fraction of adsorption removal decreased dramatically; between pH 7.12 and pH 12.2, it decreased marginally. The best dye adsorption at pH 1.5 (protonation of sorbent sites–H^+^) was caused by the electrostatic interaction between the negatively charged anionic dye molecules and the positively charged MBCOT surface sites. Nevertheless, as the system's pH rose, the proportion of AR73 dye molecules adsorbed to the adsorbent dropped because the MBCOT (^–^OH) sites were deprotonated. The negatively charged anionic AR73 dye molecules and the negatively charged areas on the MBCOT surface were repelled by electrostatic forces as a result. Because of the adsorbent (MBCOT) surface properties and the ionisation or dissociation of the sorbate (AR73 dye) molecule, the pH of the system also affects the sorptive adsorption of sorbate molecules. It is possible to ascertain the pH at which the adsorbent surface demonstrates net electrical neutrality using the point of zero charge (pH_PZC_). The pH_PZC_ was discovered to be 10.7, as seen in Fig. 2b. The sites on the sorbent surface were positively charged if the pH was less than the pHPZC; otherwise, they were negatively charged. These results are in line with our previous study^61^.

#### Effect of contact time

**Figure 3:**
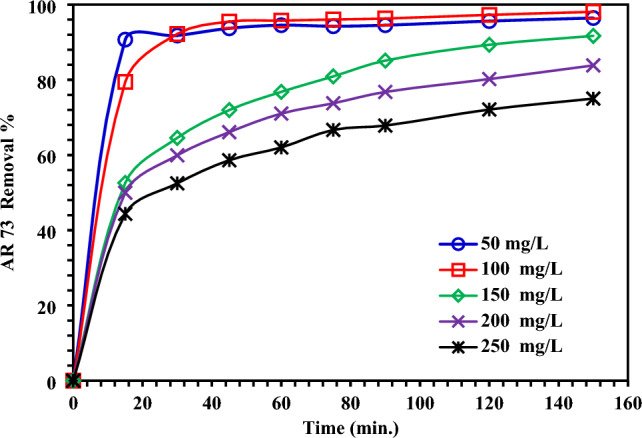
Impact of contact time on the AR73 dye removal with MBCOT adsorbent (50–250 mg/L initial AR73 dye concentration, 1.5 g/L MBCOT adsorbent dose, temperature = 25 ± 2 °C).

Equilibrium time is one of the most important properties of inexpensive adsorption systems. The brief equilibrium duration demonstrates that adsorption was effective right away. When the pH was 1.5, the initial AR73 dye concentrations varied from 50 to 250 mg/L, and the MBCOT adsorbent concentration was 1.5 g/L, Fig. 3 shows the time-dependent elimination of the dye. After the first 15 min, the adsorption process picks up speed and increases steadily. Figure 3 demonstrates that the first 15–30 min account for roughly 79.44–92.19% of the adsorption of AR73 dye.

As the contact duration increased, the quantity of AR73 dye that was removed grew steadily. After 2.5 h, the removal was 96.44, 98.08, 91.69, 83.83, and 75%, respectively, depending on the starting concentration (50–250 mg/L). Most of these ions will be able to cling to the adsorbent because of the low dye concentration of the empty active sites in the removal of AR73 dye with a low beginning concentration of the MBCOT adsorbent. However, the removal percentage of AR73 dye will stay low when a high initial MBCOT adsorbent concentration is employed because the empty active sites are limited in their ability to adsorb further dye after being loaded with a certain amount of AR73 dye. The adsorption process occurs quickly and proves to be cost-effective in industrial applications.

#### Impact of initial AR73 dye concentration

**Figure 4:**
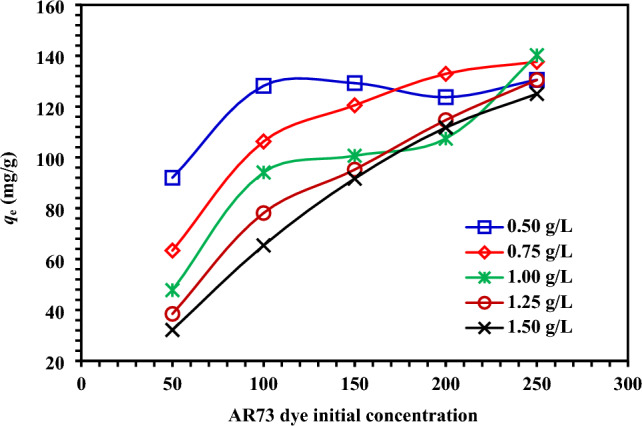
Impact of initial AR73 dye concentration (50–250 mg/L) on *q*_e_ (mg/g) using MBCOT at various dosages (0.5–1.5 g/L).

To investigate the effect of the initial concentration of AR73 dye on the adsorption capacity at equilibrium (*q*_e_), it is essential to determine the initial concentration of the adsorbed material. The effects of MBCOT dose on equilibrium adsorption capacity (*q*_e_), 0.5–1.5 g/L MBCOT concentration and 50–250 mg/L initial AR73 dye concentration were studied at 25 °C and solution pH 1.5. The *q*_e_ quantity of AR73 dye adsorbed at the same starting concentration of AR73 dye was found to rise at equilibrium as MBCOT doses dropped, as shown in Fig. 4. Using MBCOT adsorbents at several dosages (0.5–1.5 g/L), the adsorption capacities at equilibrium (*q*_e_) in eliminating AR73 dye were established 50, 100, 150, 200, and 250 mg/L initial AR73 dye concentrations, the *q*_e_ values vary from 92.05 to 32.15, 128.9 to 65.39, 129.25 to 91.69, 123.73 to 111.77, and 130.54 to 125.01 mg/g, respectively.

The adsorption capacity (*q*_e_) of AR73 dye on MBCOT is influenced by the starting concentration of the dye in the equilibrium state, as shown in Fig. 4. It was discovered that *q*_e_ dropped as the adsorbent dose rose. Thus, the initial concentration of AR73 dye dictated how well it adsorbs from its aqueous solution. This conclusion is supported by similar research in the literature^33,44^. The boundary layer effect is the first thing that AR73 dye molecules experience when they adsorb on MBCOT adsorbent. The molecules then diffuse from the boundary layer film to the surface of the adsorbent, where their porous nature finally causes them to adhere.

#### Impact of MBCOT dosage on AR73 dye adsorption

**Figure 5:**
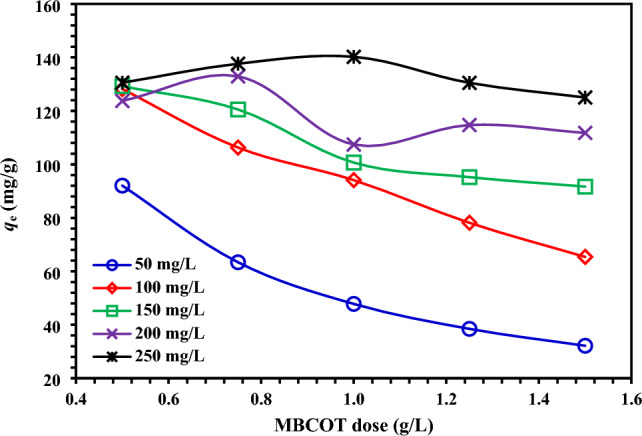
Effect of different MBCOT doses on the removal of AR73 dye (50–250 mg/L initial AR73 dye concentration, pH = 1.5, Temperature = 25 ± 2 °C).

The adsorbent dosage is a significant aspect in determining the total cost, as well as the recycling and reuse costs, of the adsorption process. As shown in Fig. 5, the effect of MBCOT dosage on AR73 dye removal was investigated by adjusting the initial AR73 dye concentration (50–250 mg/L), MBCOT dosages (0.5–1.5 g/L), solution pH to 1.5, temperature (25 ± 2 °C), and contact time (150 min). While AR73 dye removal reached over 74% removal within 15 min, it gradually increased with advancing contact time. The maximum AR73 dye removal percentage and the lowest adsorption quantity at equilibrium (*q*_e_) were discovered using a 1.5 g/L MBCOT dosage.

### Adsorption ısotherms

**Figure 6:**
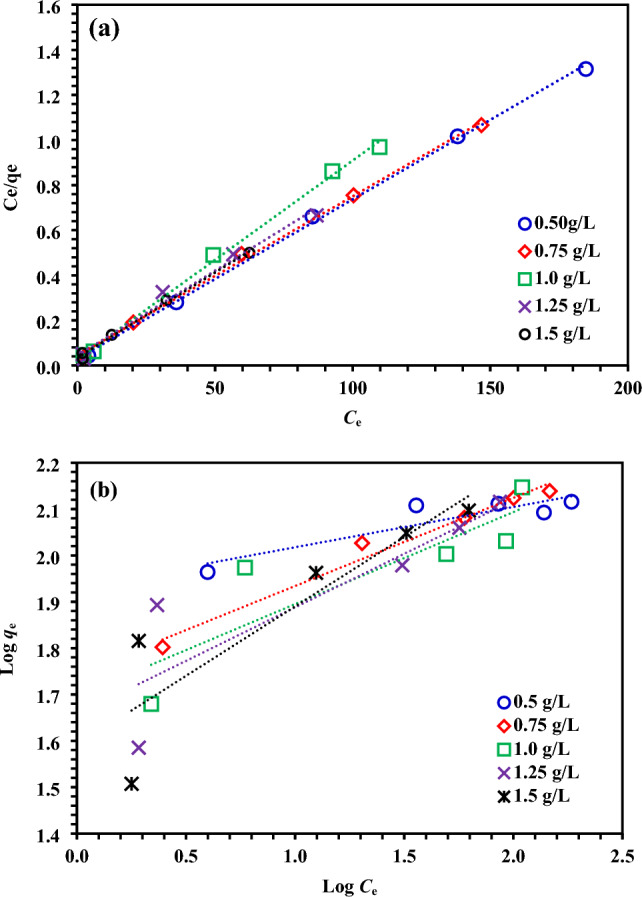
(**a**) Langmuir; and (**b**) Freundlich isotherms for AR73 dye of initial concentrations (50–250 mg/L) onto MBCOT doses (0.5–1.5 g/L) at 25 ± 2 °C.

In this step of the study, the MBCOT adsorbent was used to adsorb AR73 solutions with different initial concentrations ranging from 50 to 250 mg/L. Equilibrium studies were conducted using 0.15 g of MBCOT dosage, a solution initial pH of 1.5, and a temperature of 25 ± 2 °C. The Langmuir and Freundlich isotherms were employed to determine the equilibrium quantities for AR73 adsorption, as shown in Fig. 6. The Langmuir isotherm model is the most well-known model for monolayer adsorption. Its equation is given in Eq. 3^65^:

3 $$\frac{{C}_{e}}{{q}_{e}}=\frac{1}{{K}_{a}{Q}_{m}}+\frac{{C}_{e}}{{Q}_{m}}$$

*q*_e_ (mg/g) adsorption capacity at equilibrium; *C*_e_ (mg/L) solution concentration at equilibrium; *Q*_m_ (mg/g) is the amount of substance adsorbed in the monolayer per unit adsorbent and *K*_a_ (L mg^–1^) is the Langmuir constant. By drawing the graph of *C*_e_/*q*_e_ against *C*_e_ from the adsorption equilibrium data, *Q*_m_ is calculated from the slope and *K*_a_ is calculated from the shift (Fig. 6a).

Heterogeneous, reversible, and multilayer adsorption is specified by the Freundlich isotherm model. Equation 4 provides the adsorption equation for the Freundlich adsorption isotherm model^66^:

4 $$\text{log}{q}_{e}=\text{log}{K}_{F}+\frac{1}{n}\text{log}{C}_{e}$$

*K*_F_ (mg^1–(1/n)^ L^1/n^ g^–1^ ) and *n* are Freundlich constants. By plotting the diagram of log *q*_e_ versus log *C*_e_, the *n* value is calculated from the slope and the *K*_F_ value is calculated from the drift (Fig. 6b).

With the help of equations for each isotherm model, AR73 adsorption isotherm constants and regression coefficients of the isotherms were established and are provided in Table 1. The regression coefficients in Table 1 show that the AR73 adsorption equilibrium data is compatible with the Langmuir isotherm model. For the Langmuir isotherm model, the adsorption capacity (*Q*_m_) in the monolayer was calculated as 140.85 mg/g. The Langmuir isotherm is consistent with the adsorption equilibrium results, which demonstrate the homogenous structure and similar active regions of the MBCOT surface.

**Table 1:** The parameters were calculated using Langmuir and Freundlich isotherm of adsorption experiments using 0.5–1.5 g/L MBCOT doses and 50–250 mg/L initial AR73 dye concentration at 25 ± 2 °C.

Isotherm model	Isotherm parameters	MBCOT doses				
		0.5 g/L	0.75 g/L	1.0 g/L	1.25 g/L	1.5 g/L
Langmuir	*Q*_*m*_ (mg/g)	140.85	140.85	113.64	133.33	131.58
	*K*_*a*_	0.237	0.163	0.314	0.170	0.222
	*R*^*2*^	0.999	0.998	0.997	0.984	0.996
Frundlich	*Q*_*m*_ (mg/g)	119.74	125.96	123.99	138.48	174.69
	*1/n*	0.087	0.190	0.199	0.231	0.230
	*K*_*F*_	85.23	55.44	49.70	45.37	38.93
	*R*^*2*^	0.819	0.977	0.762	0.757	0.788

### Adsorption Kinetic Studies

Using data on AR73 adsorption, the kinetic behaviour of the adsorption process was tried to be elucidated. Kinetic studies were conducted using the pseudo-first-order (PFO), pseudo-second-order (PSO), film diffusion (FD), and intraparticle diffusion (IPD) models. The equations of the PFO kinetic model developed by Lagergren^67^ and the PSO kinetic model developed by Ho and Mckay^68^ are given in Eqs. 5 and 6.

5 $$\text{log }({q}_{e}-{q}_{t})=\text{log }{q}_{e}-\frac{{k}_{1}}{2.303}t$$

6 $$\left(\frac{t}{{q}_{t}}\right)=\frac{1}{{k}_{2}{q}_{e}^{2}}+\frac{1}{{q}_{e}}(t)$$

**Figure 7:**
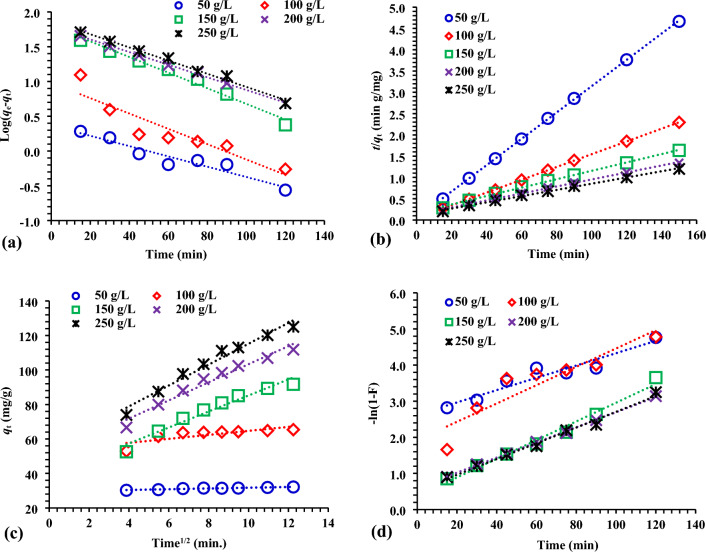
The diagrams of (**a**) PFO; (**b**) PSO; (**c**) IPD; (**d**) FD models with starting AR73 dye concentrations of 50–250 mg/L and 1.5 g/L MBCOT doses at 25 ± 2 °C.

*q*_e_ and *q*_t_ (mg/g) are the adsorption capacity at equilibrium and at time *t*, respectively, and *k*_1_ (min^-1^) and *k*_2_ (g mg^–1^ min^–1^) are constants belonging to PFO and PSO kinetic models, respectively. For the PFO kinetic model, the graph of ln (*q*_e_–*q*_t_) against *t* is drawn to find the constant qe from the slip value and *k*_1_ from the slope value (Fig. 7a). In the PSO kinetic model, the graph of *t*/*q*_t_ against *t* is drawn and the constant qm is calculated from the slope and *k*_2_ is calculated from the shift (Fig. 7b).

Kinetic constants calculated using the data in Fig. 7 are as given in Table 2. As seen in Table 2, it was found more appropriate to represent the adsorption data with a PSO kinetic model. Equation 7 determines the diffusion coefficient in the IPD model^69^.

7 $$ q_{t}  = K_{{dif}} t^{{{\raise0.7ex\hbox{$1$} \!\mathord{\left/ {\vphantom {1 2}}\right.\kern-\nulldelimiterspace} \!\lower0.7ex\hbox{$2$}}}}  + C $$

where *C* is the intercept and *K*_dif_ is the IPD rate constant (mg g^–1^ min^1/2^) and the constant related to the boundary layer thickness (mg/g), respectively (Fig. 7c). The intercept *C* values give information regarding the thickness of the boundary layer; as the intercept increases, so does the resistance to external mass transfer^70^. The liquid FD model^71^ can be used (Eq. 8) when the solute molecules' passage from the liquid phase to the solid phase boundary is the most important factor in adsorption^72,73^.

8 $$\text{ln}\left(1-F\right)=-{K}_{FD}(t)$$

where *F* and *K*_FD_ are the fractional attainments of equilibrium (*F* = *q*_t_/*q*_e_), and the FD rate constant, respectively (Fig. 7d). Due to the parameters calculated in Tables 2 and 3, the PSO model best suited the experimental data because of the final value of the linear regression coefficient (*R*^2^) achieved (> 0.990).

**Table 2:** PFO and PSO model results by 0.25–1.25 g/L MBCOT doses and 50–250 mg/L starting AR73 dye concentration at 25 ± 2 °C.

Parameter			First-order kinetic model			Second-order kinetic model			
MBCOT Conc	AR73 dye (mg/L)	*q*_*e*_ (exp.)	*q*_*e*_ (calc.)	*k*_*1*_	*R*^*2*^	*q*_*e*_ (calc.)	*k*_*2*_ × 10^3^	*h*	*R*^*2*^
0.75 g/L	50	92.05	59.10	31.78	0.937	99.01	0.91	8881	0.999
	100	128.19	70.05	18.88	0.987	138.89	0.41	7974.5	0.994
	150	129.25	66.77	23.03	0.944	140.85	0.23	4564.1	0.959
	200	123.73	104.35	28.10	0.914	138.89	0.36	6973.5	0.996
	250	130.54	98.97	20.73	0.890	151.52	0.23	5382.1	0.980
1.0 g/L	50	63.38	24.23	33.16	0.952	65.79	2.61	11,299.4	0.999
	100	106.29	65.45	27.41	0.964	114.94	0.64	8431.7	0.998
	150	120.52	104.71	35.24	0.872	133.33	0.25	4454.3	0.966
	200	132.92	81.34	20.04	0.993	147.06	0.35	7501.9	0.995
	250	137.64	100.53	17.96	0.950	156.25	0.30	7385.5	0.993
1.25 g/L	50	47.81	11.88	30.40	0.978	49.02	5.18	12,437.8	1.000
	100	94.11	60.62	28.79	0.898	101.01	0.79	8051.5	0.998
	150	100.67	62.85	21.42	0.979	111.11	0.47	5861.7	0.997
	200	107.42	94.49	19.81	0.990	136.99	0.16	3088.3	0.997
	250	140.23	87.32	19.11	0.988	142.86	0.46	9478.7	0.996
1.50 g/L	50	38.47	4.46	22.80	0.954	38.91	11.53	17,452.0	1.000
	100	78.14	31.92	29.94	0.986	81.97	1.69	11,325.0	1.000
	150	95.22	56.31	20.04	0.979	105.26	0.44	4890.0	0.992
	200	114.72	66.82	19.11	0.998	126.58	0.41	6605.0	0.997
	250	130.49	79.60	19.81	0.995	144.93	0.35	7326.0	0.996
1.75 g/L	50	32.15	2.33	17.04	0.920	32.36	18.50	19,379.8	1.000
	100	65.39	9.57	25.33	0.847	66.67	5.14	22,831.1	1.000
	150	91.69	63.18	25.79	0.983	101.01	0.58	5903.2	0.999
	200	111.77	61.14	20.96	0.999	121.95	0.52	7704.2	0.998
	250	125.01	73.88	21.88	0.989	136.99	0.43	8123.5	0.998

**Table 3:** IPD ana FD model results by 0.25–1.25 g/L MBCOT doses and 50–250 mg/L starting AR73 dye concentration at 25 ± 2 °C.

MBCOT Conc	AR73 dye (mg/L)	Interaparticle diffusion			Film diffusion	
		*K*_*dif*_	*C*	*R*^*2*^	*K*_*FD*_	*R*^*2*^
0.5 g/L	50	3.491	52.730	0.947	0.032	0.937
	100	5.875	57.660	0.988	0.019	0.987
	150	7.767	28.170	0.931	0.023	0.944
	200	6.559	48.004	0.981	0.028	0.914
	250	7.896	36.326	0.960	0.021	0.890
0.75 g/L	50	1.384	47.792	0.923	0.033	0.952
	100	4.629	53.792	0.956	0.027	0.964
	150	7.048	29.452	0.931	0.035	0.872
	200	6.850	52.167	0.982	0.020	0.993
	250	7.580	50.781	0.970	0.018	0.950
1.0 g/L	50	0.798	38.910	0.913	0.002	0.895
	100	3.765	50.886	0.963	0.029	0.898
	150	5.274	39.350	0.943	0.021	0.979
	200	8.367	9.673	0.959	0.020	0.990
	250	6.374	58.790	0.919	0.019	0.988
1.25 g/L	50	0.337	34.519	0.985	0.023	0.954
	100	2.387	51.965	0.852	0.030	0.986
	150	5.045	34.252	0.968	0.020	0.979
	200	5.985	44.475	0.969	0.019	0.998
	250	6.836	50.097	0.978	0.020	0.995
1.5 g/L	50	0.219	29.530	0.931	0.017	0.920
	100	1.155	53.137	0.636	0.025	0.847
	150	4.645	38.785	0.959	0.026	0.983
	200	5.248	50.848	0.964	0.021	0.999
	250	6.055	54.657	0.971	0.022	0.989

The PSO model demonstrated that the calculated and experimentally determined *q*_e_ values were remarkably close, indicating that the model could accurately describe the absorption process of AR73 dye into MBCOT. Redefining steps and diffusion did not have an impact on the IPD and FD linear plots via the liquid layer surrounding the sorbent since the linear plots did not continue through the origin of their respective plots. In addition, the IPD and FD models' *R*^2^ values were less than the PSO model's. Thus, the absorption of AR73 dye to MBCOT sorbent was supported by the notion of chemisorption involving valency force via electron sharing or exchange between MBCOT and AR73 dye molecules.

### Regeneration of MBCOT

**Figure 8:**
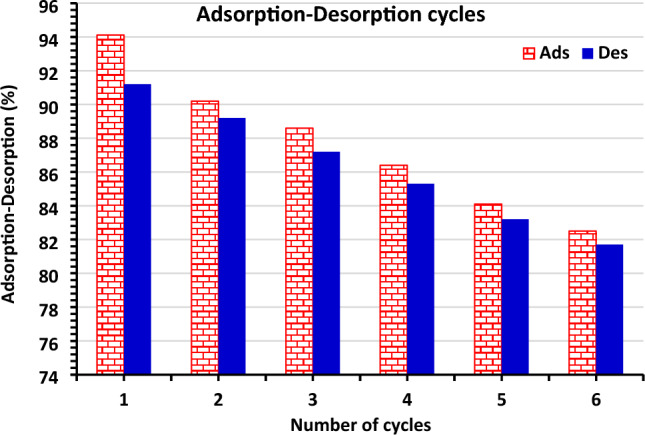
The regeneration cycles effect of MBCOT using 0.1 M NaOH and 0.1N HCl showing the percentage of AR73 dye that was desorption as well as the AR73 dye adsorption cycles using 1.0 g/L MBCOT and 100 mg/L of AR73 dye concentrations.

The viability and reusability of MBCOT for AR73 dye absorption were investigated by performing desorption studies of the dye using 0.1 M NaOH and HCl as an extraction environment. As may be shown in Fig. 8, the study's percentage of AR73 dye desorption rose and then decreased with regeneration cycles. Six cycles of absorption and desorption were examined using regeneration MBCOT. Throughout the cycles, the change in adsorption and desorption was generally consistent. But after six cycles, it dropped by around 10%. It means that it can be used to remove AR73 dye from water.

### Comparison of MBCOT with other adsorbents in AR73 dye removal

There are few studies using mandarin peel biochar as dye removal or adsorbent, and there is no research on AR73 dye adsorption using mandarin peel biochar. As a result, comparable research from the literature is included in Table 4, where it is presented that MBCOT adsorbent is successful in removing AR73 dye molecules from its water solution.

**Table 4:** Comparing MCO_2_T with different used materials for the removal of AR73 and different dyes.

Adsorbents	Dye	*Q*_m_(mg/g)	References
Carbon-Based Mandarin Orange Peels	Methylene blue	74.15	^8^
Mandarin biochar	Methylene blue Basic fuchsin	99.11 78.01	^74^
Mandarin peel biochar	Methyl Orange Fast Green	16.27 12.44	^75^
Modified *Citrus reticulata* peels	Acid Yellow 73	96.46	^76^
Copper diethyldithiocarbamate Copper dimethyldithiocarbamate	AR73	42.9 37.8	^77^
*Parthenium hysterophorus L*	AR73	2.86	^78^
Rice Wine Lees	AR73	18,74	^79^
HCl-water hyacinth stems biomass	AR73	50.0	^80^
Polyacrylamide-titanium dioxide	Crystal Violet	38.9	^81^
Glutaraldehyde cross-linking chitosan/fluorapatite	Methyl Orange	225.55	^82^
MBCOT	AR73	140.85	This Study

### Adsorption mechanism of AR73 Dye by MBCOT

**Figure 9:**
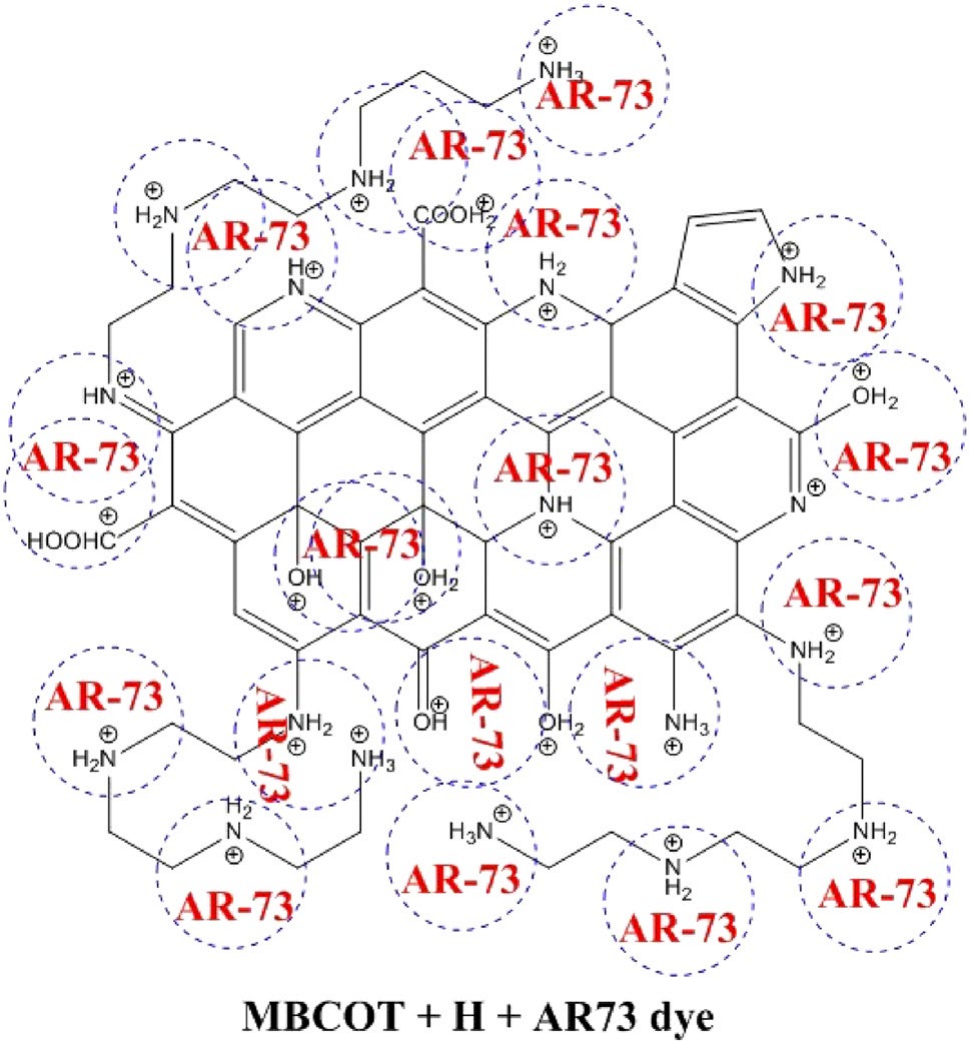
The adsorption mechanism of AR-73 dye by a possible structure of MBCOT adsorbent in acidic medium.

Figure 9 explains the likely adsorption mechanism via which MBCOT adsorbed the AR73 dye ions. According to FTIR analysis, a variety of functional groups, including C=O, –NH, hydroxyl O–H, C=N–, C–O–H and NH_2_ groups, developed on the surface of the MBCOT. Because of the electrostatic interaction between the nitrogen and oxygen lone pair on the MBCOT surface and the negative charge on the AR73 dye, the adsorption mechanism of the AR73 dye ions in an acidic medium (pH 1.5) can be accomplished through physical interaction. In an acidic environment, the surface of MBCOT picks up a positive charge, which attracts negatively charged dye molecules^26,83^. Furthermore, there is interaction between the functional groups of the positive ions on the MBCOT's surface and the negative ions in the solution. The dye molecules are more soluble at an acidic pH, which makes it easier for them to adhere to the adsorption sites and diffuse through the MBCOT's pores. Since the acidic pH of MBCOT is necessary to promote the adsorption of AR73 dye molecule onto the material surface, it is a great way to remove colour from industrial effluent. The most important mechanism is the electrostatic interaction-mediated adsorption of ionizable organic molecules to the positively charged surface of the adsorbents^84^. An aqueous solution's pH and ionic strength determine how well it draws or repels contaminants^84,85^.

Moreover, the capacity of organic contaminants in industrial effluent to be adsorbed is influenced by the pH of the solution^86^. Parshetti et al.'s study^87^ examined the use of food waste-derived biochar in the adsorption of cationic dyes from wastewater. They found that an alkaline pH enhanced the adsorption of cationic dyes. It was explained by the strong interaction between the positively charged cationic dyes and the negatively charged sites of the adsorbent surface^87^. It was less successful in adsorbing cationic dyes, though, because there was an excess of H^+^ at pH 1.5, which competed with the positive charges of the cationic dye^87^. The capacity of organic and inorganic pollutants from industrial effluent to be adsorbed on biochar is hence influenced by the pH of the solution, which also affects the charged sites^88–97^. Tsai and Chen^98^ and Xu et al.^99^ have noted that pH has an impact on biochar's capacity to absorb materials.

## Conclusion

In this study, we developed an adsorbent material using biochar obtained from mandarin peels. We tested the biochar (MBCOT) from mandarin peels for its ability to adsorb AR73. We examined various operating parameters such as pH, initial concentration of AR73 dye, MBCOT adsorbent dosage, and contact time in the adsorption process. The main findings are listed below:

- The maximum removal of 98.08% was achieved at 25 ± 2 °C, pH of 1.5, 100 mg/L AR73 dye concentration, 1.5 g/L MBCOT adsorbent dose, and 150 min contact time.
- The PSO kinetic model matched the adsorption kinetics, and the most suitable Langmuir isotherm was the adsorption isotherm.
- The MBCOT adsorbent has a maximum adsorption capacity of 140.85 mg/g. Therefore, biochar obtained from mandarin peels (MBCOT) can be used as an effective adsorbent for the removal of AR73 dye in aqueous solutions.
- The derived thermodynamic parameters and isotherm model can be useful for the commercial removal of AR73 dye.

### Supplementary Information

Supplementary Information.

## Data Availability

The corresponding author can provide access to the datasets used in this investigation upon request.
