# Supplementary material for: Mandarin biochar-CO-TETA was utilized for Acid Red 73 dye adsorption from water, and its isotherm and kinetic studies were investigated
Source: Sci Rep. 2024 Jun 6;14:13021. doi: 10.1038/s41598-024-62870-x (PMC11156941; doi:10.1038/s41598-024-62870-x)
Supplement: Supplementary file 1 — Supplementary Information. [file 41598_2024_62870_MOESM1_ESM.docx]

**Supplementary data**

**Mandarin biochar-CO-TETA was utilized for Acid Red 73 dye adsorption from water, and its isotherm and kinetic studies were investigated**

Ahmed Eleryan^1^, Eda Keleş Güner^2^, Mohamed Hassaan^1^, Mohamed A. El-Nemr^3^, Safaa Ragab^1^, Ahmed El Nemr^1^*


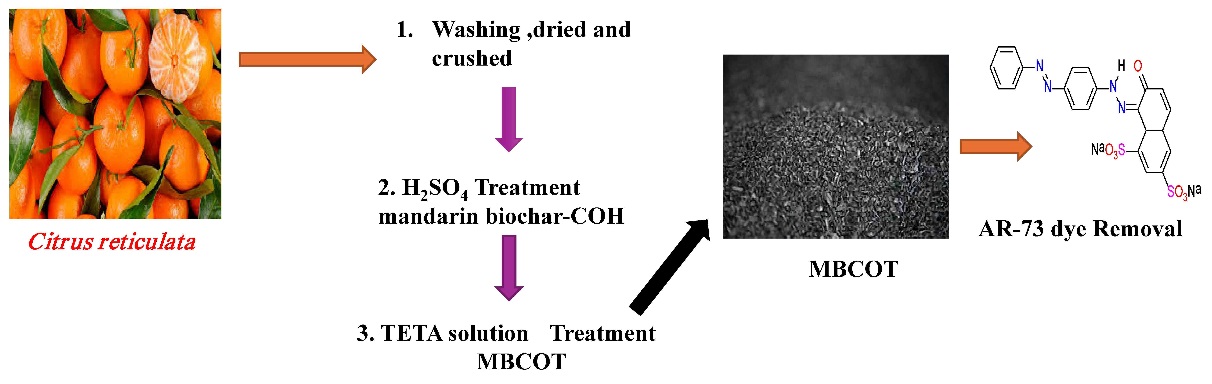


Figure S1. The synthesis procedures of the MBCOT adsorbent.


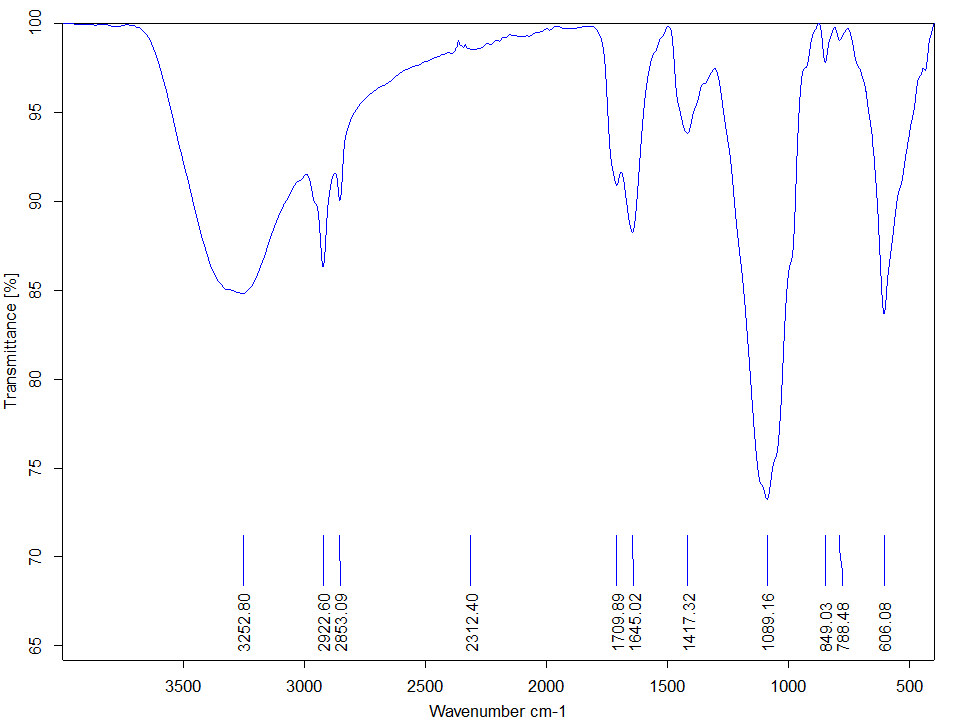


Figure S2. FTIR analysis of dry Mandarin peels.


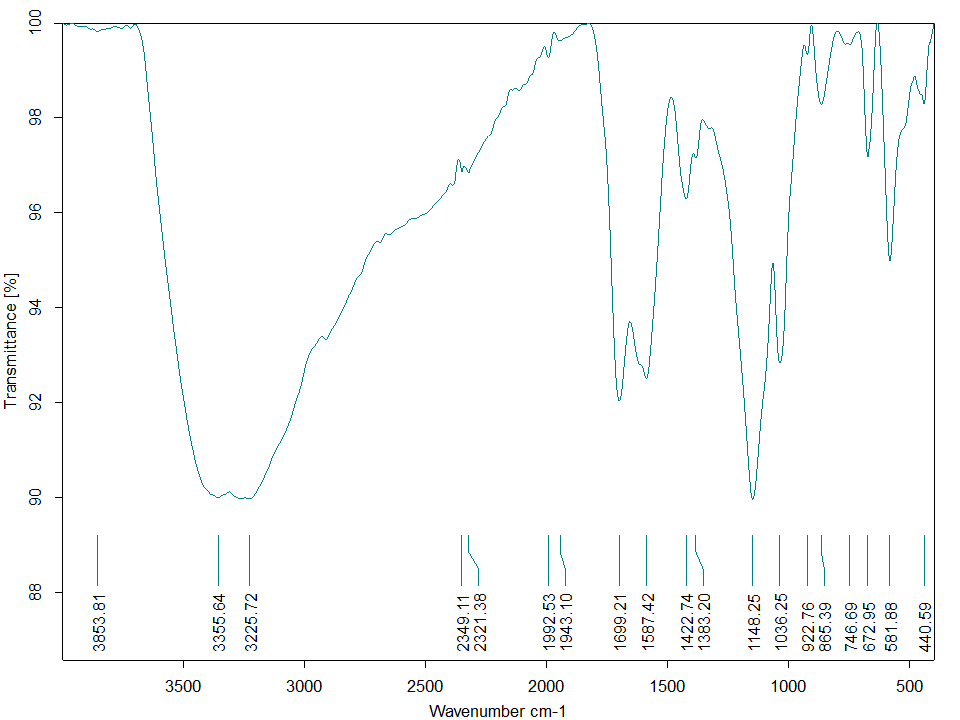


Figure S3. FTIR analysis of dry Mandarin peels Biochar.


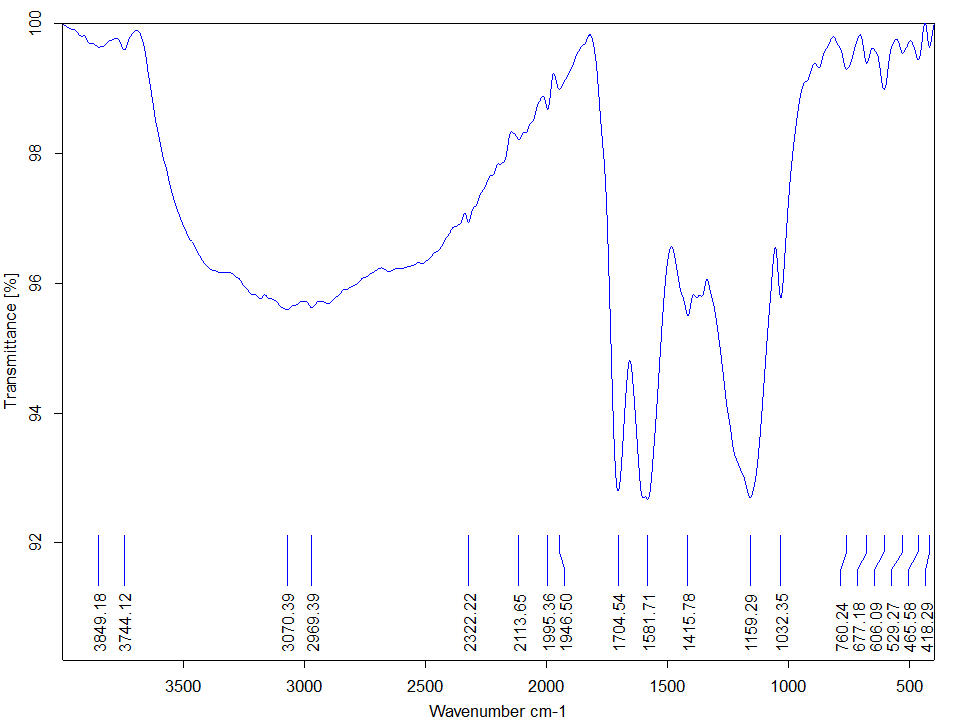


Figure S4. FTIR analysis of dry Mandarin peels Biochar-CO.


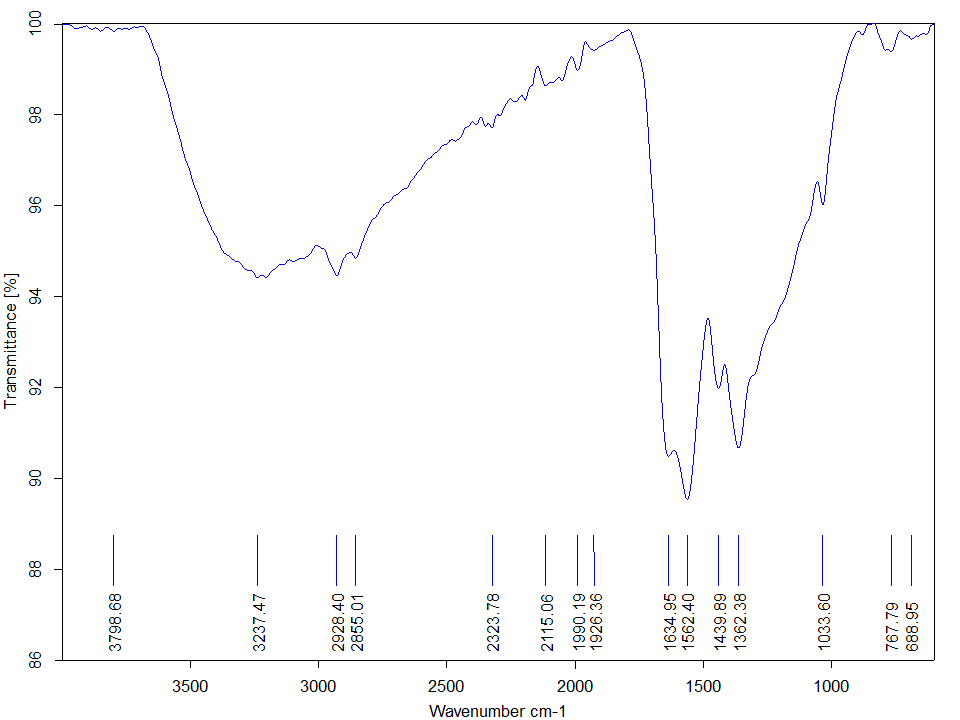


Figure S5. FTIR analysis of dry Mandarin peels Biochar-CO-TETA.


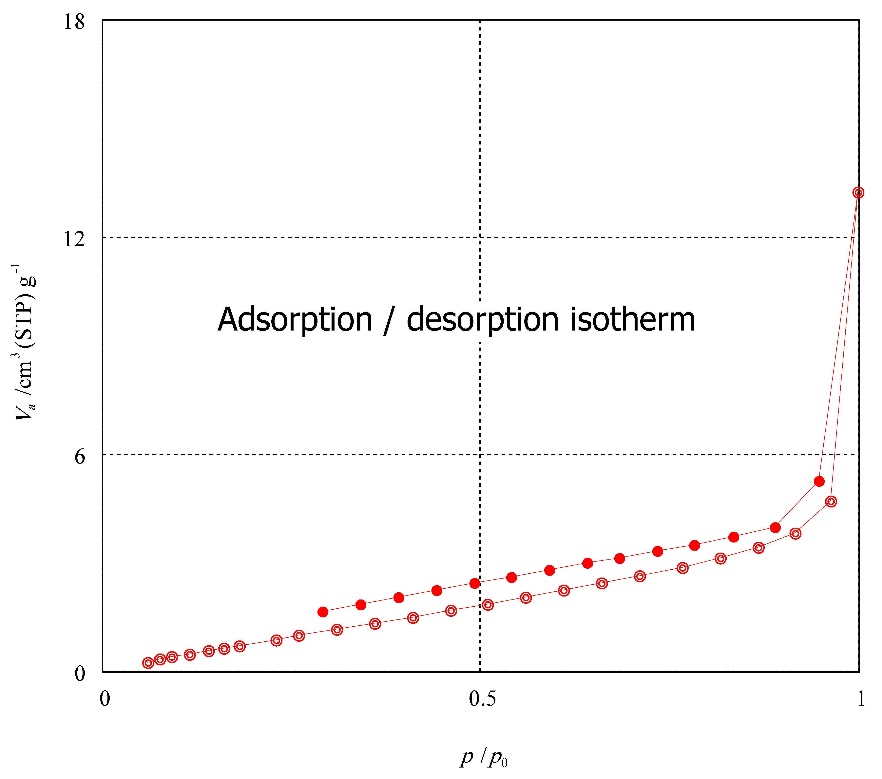


Figure S6. Adsorption/desorption isotherm analysis of Mandarin peels Biochar-CO-TETA.


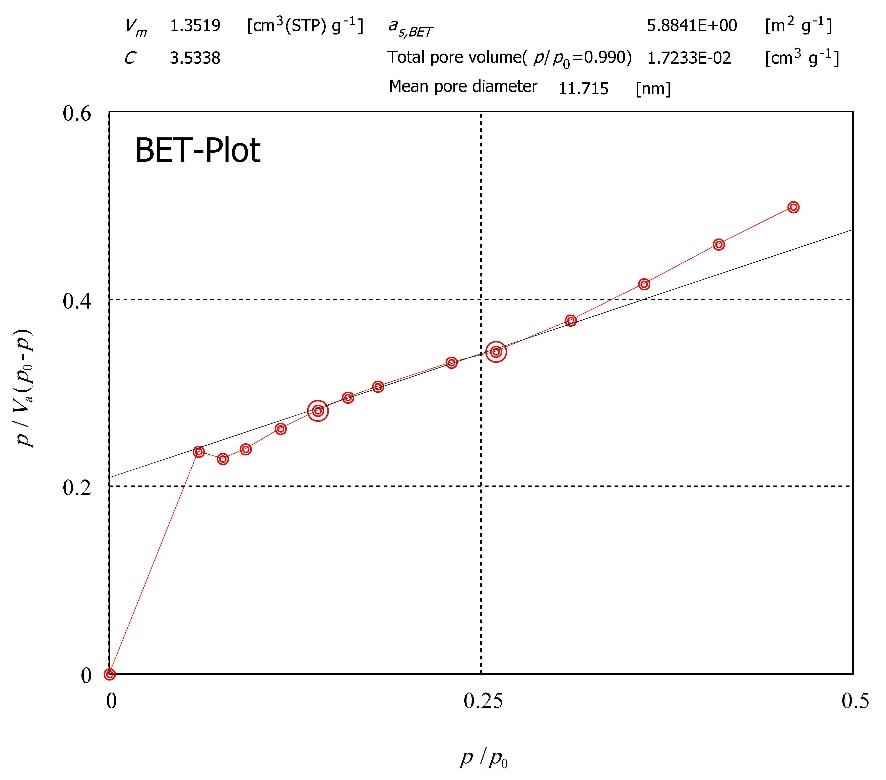


Figure S7. BET analysis of Mandarin peels Biochar-CO-TETA.


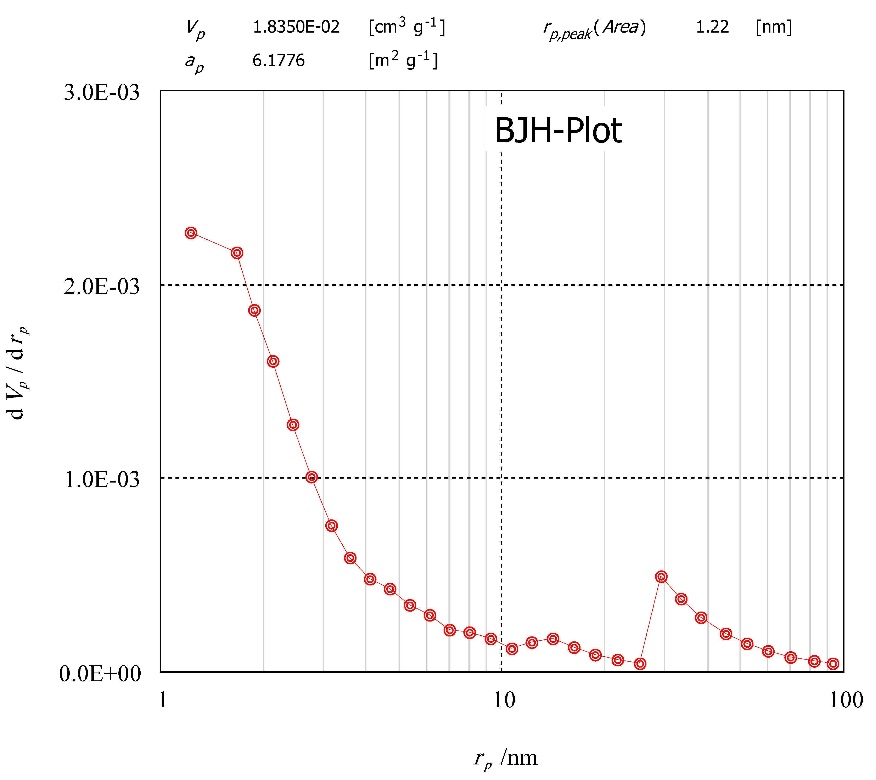


Figure S8. BJH-Plot of adsorption analysis of Mandarin peels Biochar-CO-TETA.


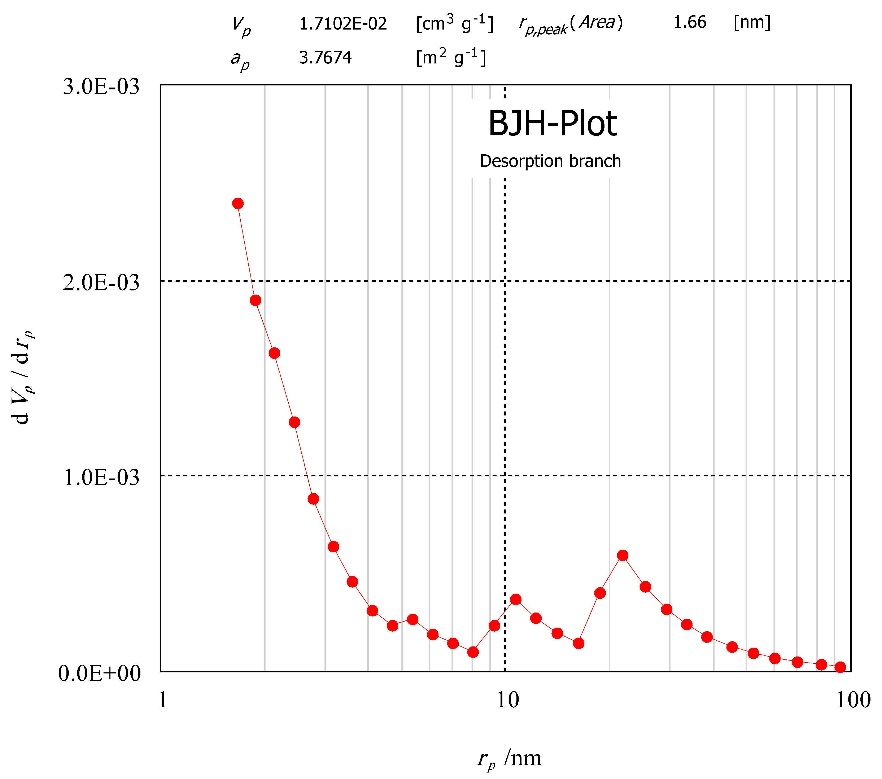


Figure S9. BJH-Plot of desorption analysis of Mandarin peels Biochar-CO-TETA.


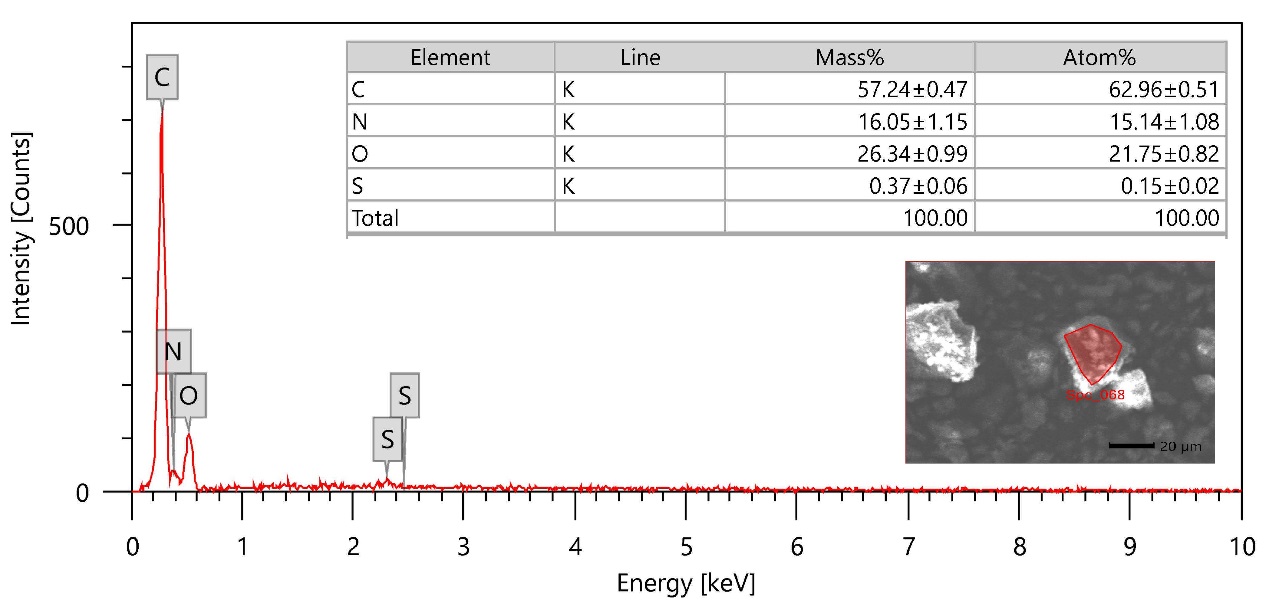


Figure S10. EDX analysis of Mandarin peels Biochar-CO-TETA.


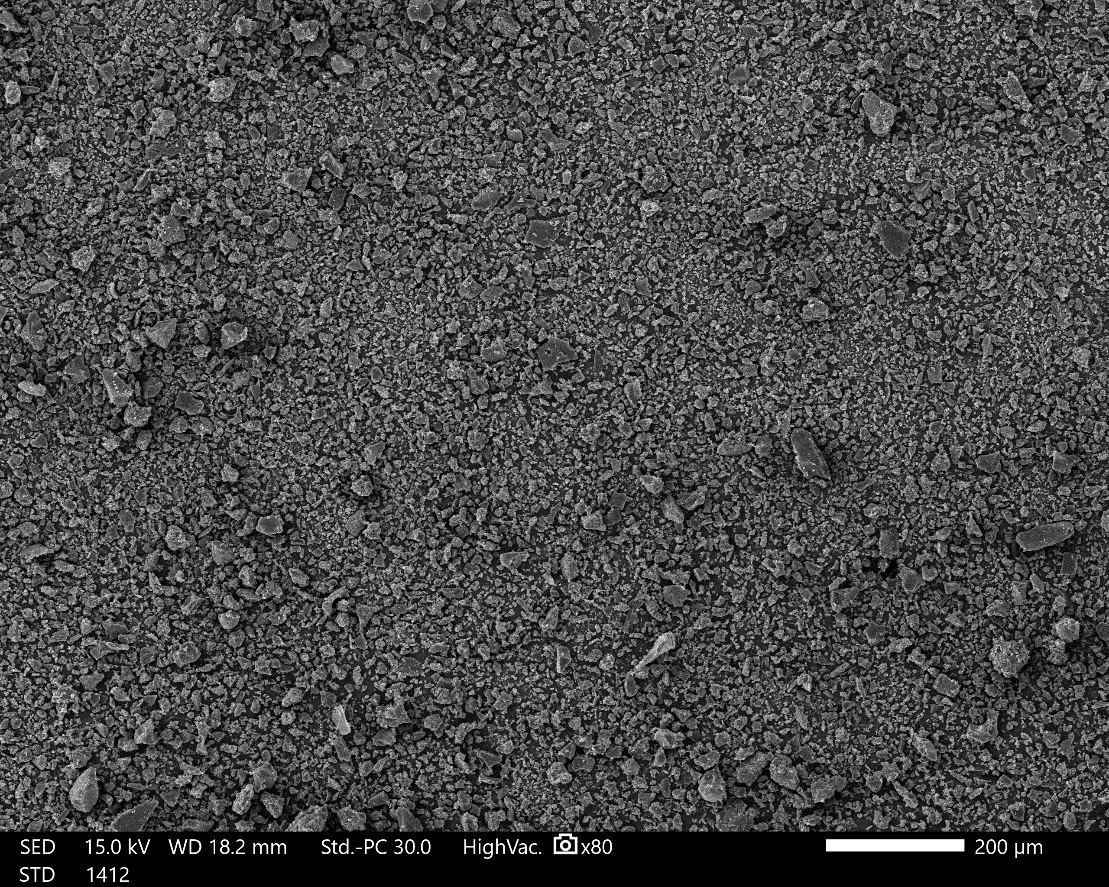


Figure S11. SEM analysis of Mandarin peels Biochar-CO-TETA.


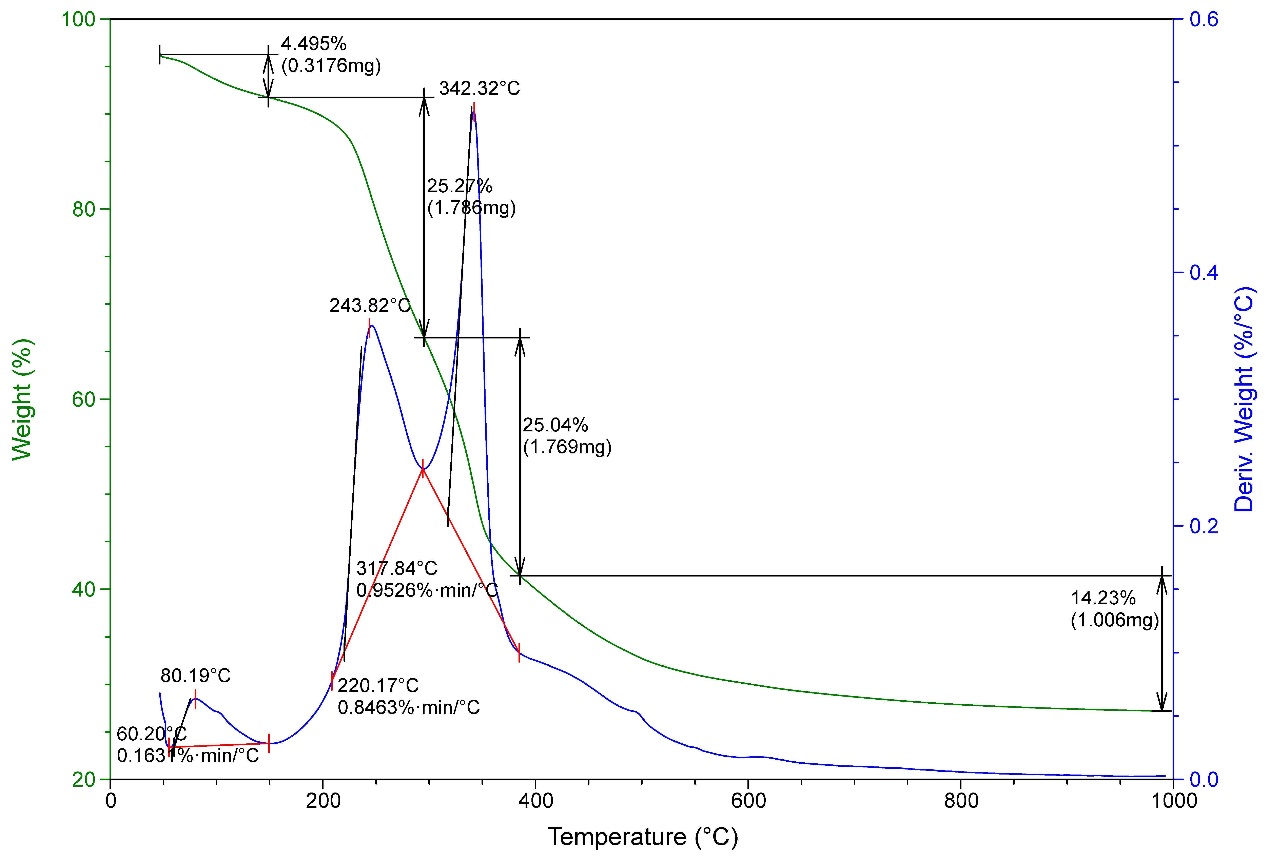


Figure S12. TGA-DTA analysis of Mandarin peels.

**
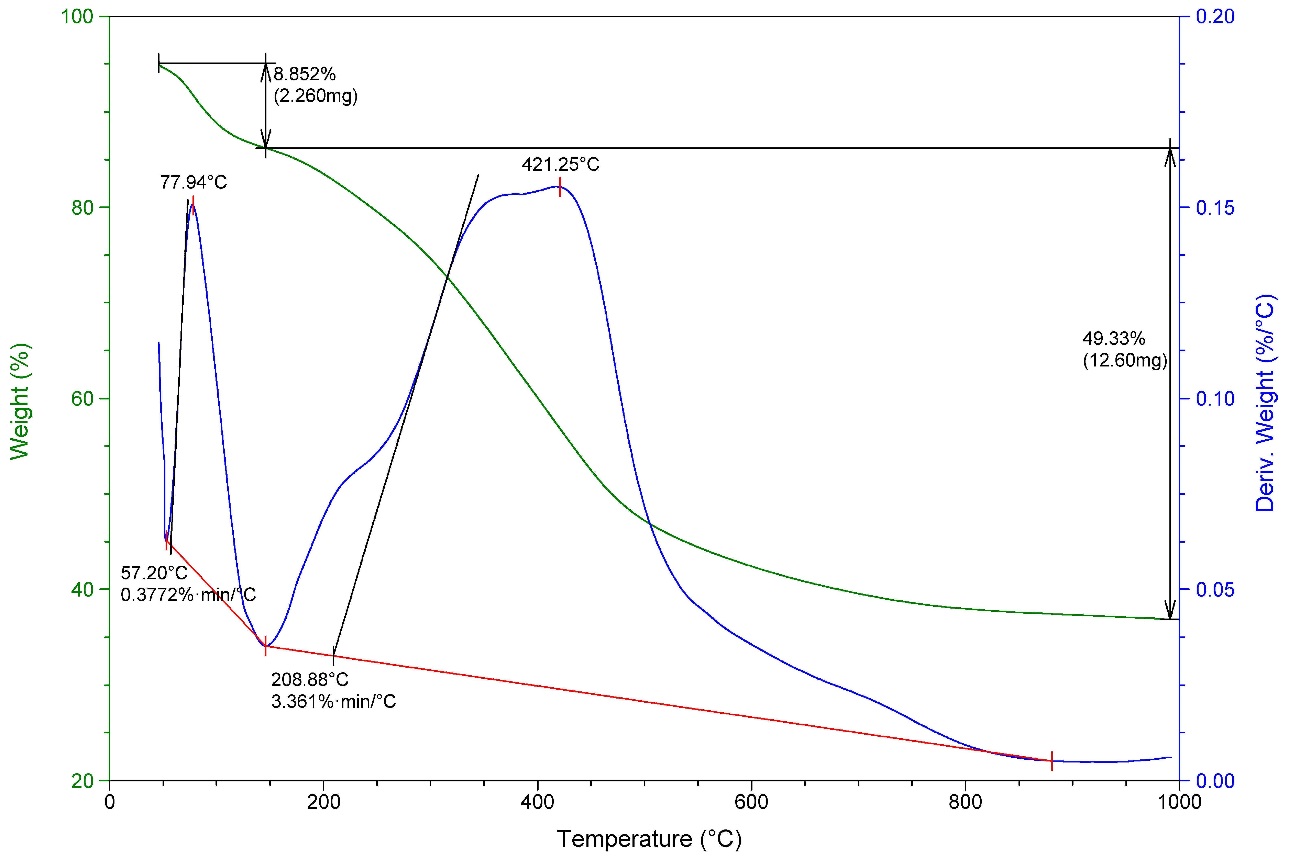
**Figure S13. TGA-DTA analysis of Mandarin peels Biochar-CO-TETA.


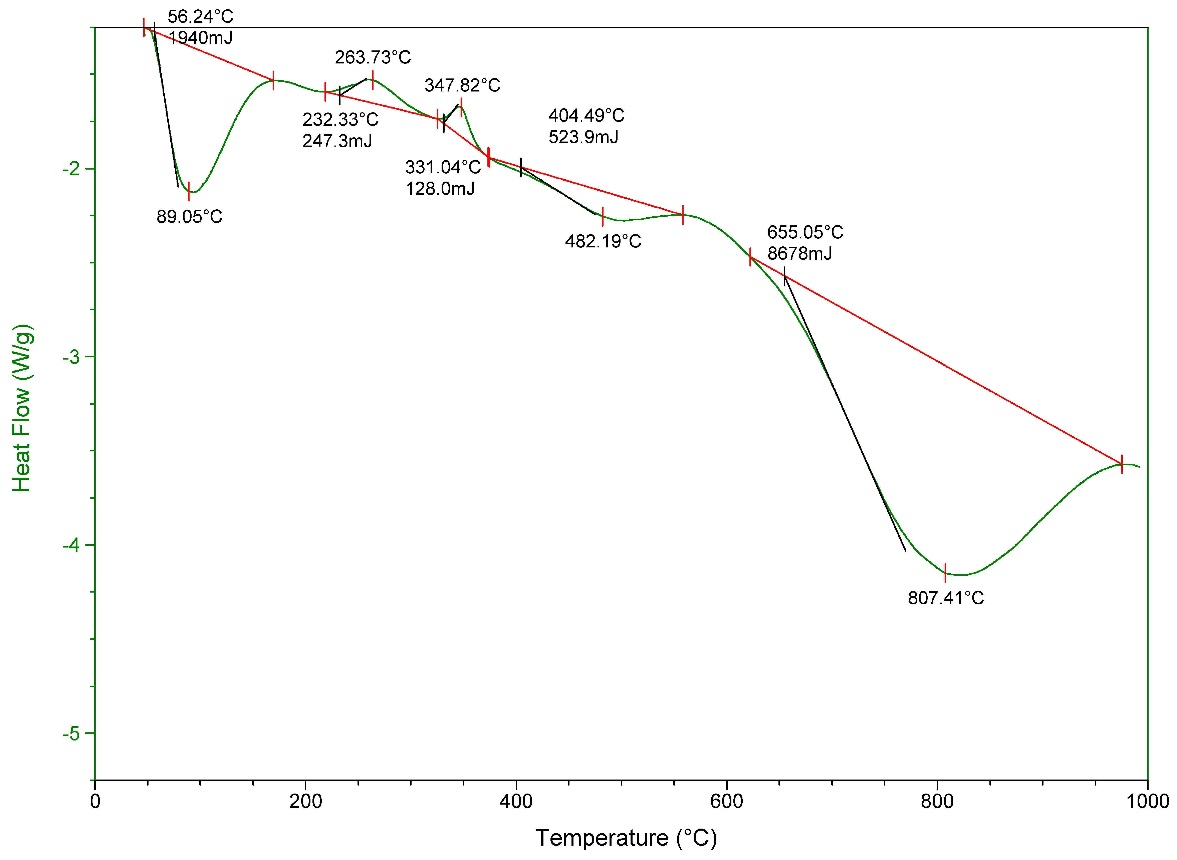


Figure S14. DAC analysis of Mandarin peels.


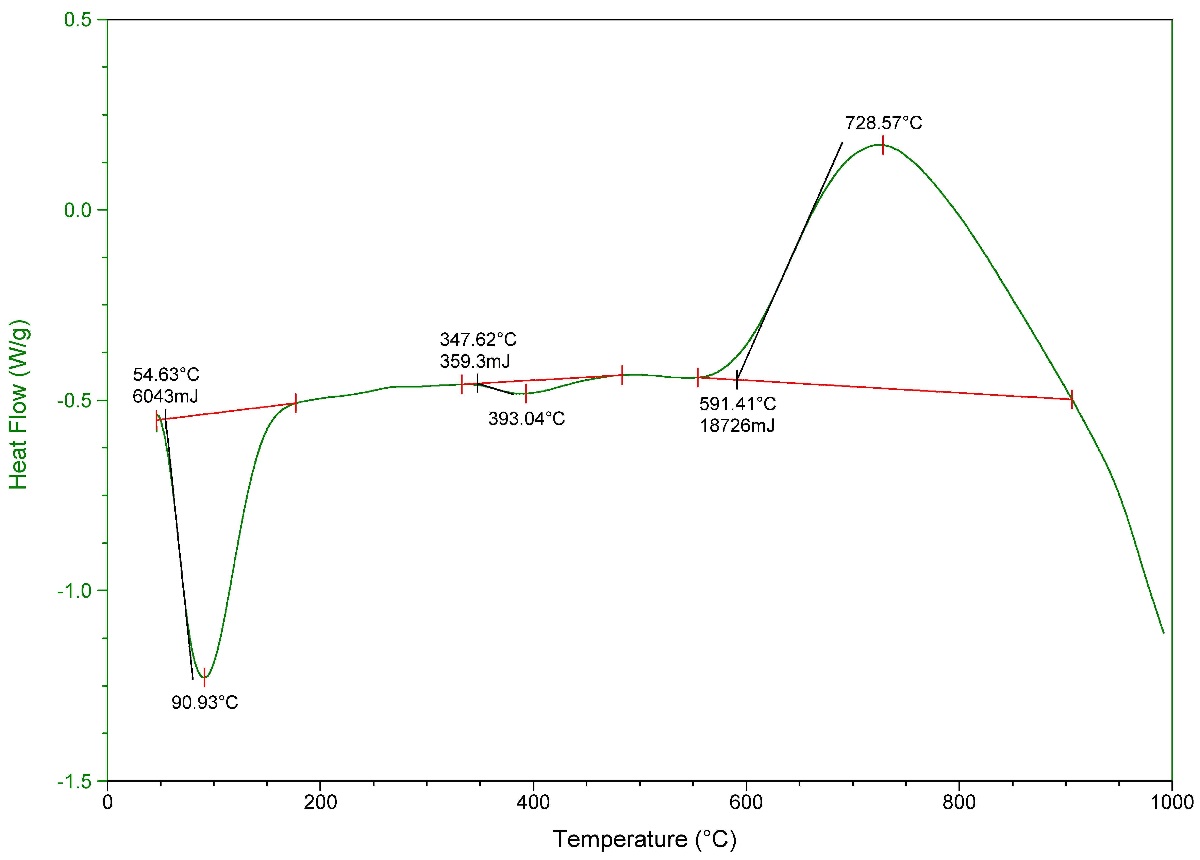


Figure S15. DSC analysis of Mandarin peels Biochar-CO-TETA.
